# Control of Rhizobia Endosymbiosis by Coupling ER Expansion with Enhanced UPR

**DOI:** 10.1002/advs.202414519

**Published:** 2025-02-22

**Authors:** Jing Ren, Qi Wang, Xiaxia Zhang, Yongheng Cao, JingXia Wu, Juan Tian, Yanjun Yu, Qingqiu Gong, Zhaosheng Kong

**Affiliations:** ^1^ State Key Laboratory of Plant Genomics Institute of Microbiology Chinese Academy of Sciences Beijing 100101 China; ^2^ University of Chinese Academy of Sciences Beijing 100049 China; ^3^ Houji Laboratory in Shanxi Province Academy of Agronomy Shanxi Agricultural University Taiyuan 030031 China; ^4^ Department of Plant Microbe Interactions Max Planck Institute for Plant Breeding Research 50829 Cologne Germany; ^5^ State Key Laboratory of Microbial Metabolism & Joint International Research Laboratory of Metabolic and Developmental Sciences School of Life Sciences and Biotechnology Shanghai Jiao Tong University Shanghai 200240 China

**Keywords:** compartmentalization, endosymbiosis, ER, symbiosomes, UPR

## Abstract

Legumes establish symbiosis with rhizobia by forming a symbiotic interface that enables cross‐kingdom exchanges of signaling molecules and nutrients. However, how host organelles interact with symbiosomes at the symbiotic interface remains elusive during rhizobia endosymbiosis. Here, symbiotic cells are reconstructed using 3D scanning electron microscopy (SEM) and uncover that the host endoplasmic reticulum (ER) undergoes dynamic expansion to gradually enwrap symbiosomes, facilitating their compartmentalization and endosymbiosis. Consistently, altering ER lamellar expansion by overexpressing *MtRTNLBs*, the reticulons responsible for ER tubulation, impairs rhizobia accommodation and symbiosome development. Intriguingly, unfolded protein response (UPR)‐marker genes, *bZIP60* and *IRE1A/B*, show continuously activated expression during nodule development, and the two UPR‐deficient mutants, *ire1b*, and *bzip60*, exhibit compromised ER biogenesis and defective symbiosome development. Collectively, the findings underpin ER expansion and UPR activation as two key events in rhizobia accommodation and reveal an intrinsic coupling of ER morphology with proper UPR during root nodule symbiosis.

## Graphical abstract

This study reconstructs legume nodule symbiotic cells using three‐dimensional (3D) scanning electron microscopy (SEM) and uncovers that endoplasmic reticulum (ER) expansion and activation of the unfolded protein response (UPR) are two key events in rhizobia accommodation. Furthermore, it reveals an intrinsic coupling between ER morphology and proper UPR function during root nodule symbiosis.

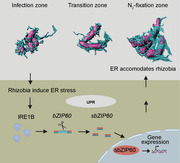

## 1 Introduction

Legumes (Fabaceae or Leguminosae) represent the third‐largest family of flowering plants. It is divided into six subfamilies. Among these, Papilionoideae and the Caesalpinioideae, contain species that enter into a root‐nodulating N_2_‐fixing symbiotic relationship with endosymbiotic rhizobia. The vast majority of the Papilionoideae subfamily is known to be nodulated, but the Caesalpinioideae is largely non‐nodulated with the exception of the tribe Mimosae (formerly the subfamily Mimosoideae or the Mimosoid clade) which is mostly nodulated.^[^ ^1^, ^2^, ^3^, ^4^ ^]^ Within legume root nodules, rhizobia differentiate into bacteroids that fix atmospheric nitrogen (N₂) into ammonia (NH₃), which is then transferred to the host plant. Bacteroids exhibit distinct differentiation patterns: in some Papilionoid legumes within the Inverted Repeat–Lacking Clade (IRLC), such as *Medicago* spp., they become swollen and terminally differentiated, whereas in legumes of the Phaseolid clade, such as soybean (*Glycine max* L.), bacteroids remain non‐swollen and retain the ability to regenerate outside the nodule.^[^ ^5^ ^]^ Among a variety of plant‐associated microorganisms, symbiotic rhizobia are striking examples of bacteria that are successfully taken up into host cells and subsequently accommodated in the membrane‐enclosed compartments within symbiotic cells.^[^ ^2^, ^3^, ^4^ ^]^ In the past twenty years, over 200 genes have been reported to be involved in symbiotic nitrogen fixation using either forward or reverse genetics approaches,^[^ ^6^, ^7^, ^8^ ^]^ which have elucidated the main signaling pathways in regulating symbiotic nitrogen fixation. However, the mechanisms underlying the uptake of rhizobia into living host plant cells and subsequent accommodation within the plant‐derived membrane system in symbiotic cells remain largely unknown, even though this process is a prerequisite for root nodule symbiosis.^[^ ^7^ ^]^

In symbiotic root nodules of the IRLC clade (and in many other studied legumes, but not all), rhizobia are entrapped into host cells via the specialized transcellular apoplastic compartments known as infection threads (ITs).^[^ ^9^, ^10^ ^]^ Subsequently, the rhizobia are taken up into symbiotic cells by an endocytosis‐like process from ITs and grow in the organelle‐like vesicles called symbiosomes.^[^ ^4^ ^]^ Along with rhizobia differentiation, symbiotic cells also undergo dramatic endoreduplication, ultimately accommodating thousands of symbiosomes to support robust nitrogen fixation.^[^ ^11^ ^]^ During symbiosome development, the symbiosome membrane undergoes massive expansion and dynamic organization, forming the symbiotic interface to achieve the accommodation of symbiotic rhizobia that efficiently fix nitrogen.^[^ ^11^, ^12^ ^]^ As early as the 1970s, electron microscopy studies have observed the presence of intracellular organelles, including the endoplasmic reticulum (ER), in symbiotic cells. Previous studies have shown that the ER participates in the formation of cytoplasmic bridges surrounding the IT tip to prompt progressive IT development.^[^ ^2^, ^9^, ^13^ ^]^ Among numerous organelles, the ER represents a multifunctional organelle harboring a wide range of structural, biosynthetic, and metabolic functions and exhibits an elaborate and flexible membrane web‐like structure spreading throughout the cytoplasm.^[^ ^14^ ^]^ However, how the host ER system contributes to symbiotic interface formation during rhizobia endosymbiosis remains to be uncovered.^[^ ^15^ ^]^

Moreover, another important function of the ER is to control the quality of proteins: only properly folded proteins are packaged into ER‐exit vesicles and allowed to move onward to their destination sites.^[^ ^16^ ^]^ In contrast, misfolded proteins are degraded by the ER‐associated degradation (ERAD) system.^[^ ^17^ ^]^ When a mismatch occurs between the load of unfolded or misfolded proteins in the ER and the capacity of the cellular machinery, it causes ER stress and further triggers the unfolded protein response (UPR).^[^ ^18^, ^19^ ^]^ The UPR is a cytoprotective response that senses an insufficiency in the ER's protein‐folding capacity and restores cellular homeostasis following physiological stress exerted on the ER. The outcomes of UPR include inhibition of mRNA translation, an increase in ER protein folding capacity, and enhanced ERAD, which are coordinated with ER membrane biogenesis to reduce the load of misfolding‐prone proteins.^[^ ^20^, ^21^ ^]^ However, although UPR plays a critical role in responding to cellular biosynthetic demands, its link to nodule symbiosis has yet to be explored. In particular, the interplay between ER morphology and UPR during nodule symbiosis is poorly understood.

In this study, we revealed that the ER forms a niche to compartmentalize and nourish symbiosomes in rhizobia‐infected cells in *Medicago truncatula* nodules. Further genetic validation confirmed that *MtRTNL*‐overexpression lines show reduced ER lamellar expansion and impaired symbiosome development. Strikingly, genes controlling UPR are continuously induced during nodule development. As expected, symbiosome development was found to be impaired in UPR‐deficient mutants, which exhibit altered ER morphology. Our findings, for the first time, uncover that ER morphological changes coordinate with enhanced UPR to achieve rhizobia accommodation and symbiosome development during root nodule symbiosis.

## 2 Results

### 2.1 ER Dynamic Expansion and Compartmentalization Regulate Symbiosome Accommodation and Development

To thoroughly explore the engagement of the host ER system in root nodule symbiosis, we performed large‐scale live‐cell imaging and observed that the ER (pseudo‐colored in red) network forms reticulate structures surrounding symbiosomes (pseudo‐colored in green) in rhizobia‐infected cells in *M. truncatula* nodules (Figure S1a, Supporting Information). We next employed scanning electron microscopy (SEM) for detailed examination. In these symbiotic cells, ER spreads extensively into the cytosol as a network of sheets and tubules accompanied by other membrane organelles (Figure S1b, Supporting Information). We then took advantage of the AutoCUTS‐SEM (Automatic collector of ultrathin sections‐SEM) technique and generated 3D‐SEM reconstruction images to show detailed ER information in a spatial manner.^[^ ^22^, ^23^, ^24^ ^]^ Interestingly, central flat ER sheets were observed to transform into cortical tubular structures to capture rhizobia (Figure S2a and Movie S1, Supporting Information). Moreover, ER tubules distributed in the periphery of symbiosomes show lumen width broadening compared to initial ER lamellar sheets (Figure S2b–e, Supporting Information).

**Figure 1:**
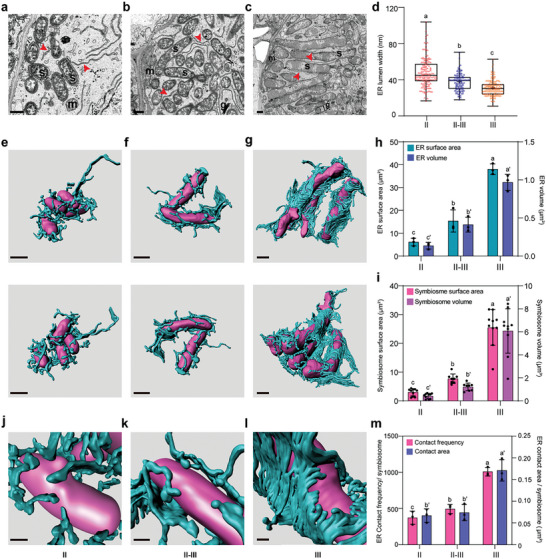
Dynamic expansion of ER surrounding the symbiosomes in *M.truncatula* nodules. a–c) Representative SEM images of 3‐week‐old nodules in zone II (a), zone II‐III (b), and zone III (c). The red arrowheads indicate ER; s, symbiosome; m, mitochondrion; g, Golgi apparatus. Scale bars = 1 µm. d) Comparison of the ER width with 2D images in different rhizobial zones (II, *n* = 150; II‐III, *n* = 118; III, *n* = 182). e–g) 3D reconstruction derived from a 50‐µm‐thick section showing that ER (blue) forms a cage‐like structure to enclose symbiosomes (magenta) in zone II (e), zone II‐III (f), and zone III (g). The vertically arranged ones formed a group, showing the interaction between ER and symbiosomes from two different perspectives. Scale bars = 1 µm. At least 10 cells were examined, and 2 cells were reconstructed in each zone with the same results. h), Quantification and comparison of the surface area and the volume of 3D symbiosomes in different nodule zones (II, *n* = 10; II‐III, *n* = 6; III, *n* = 10). i, Quantification and comparison of the surface area and the volume of the 3D ER system surrounding the individual symbiosome in different nodule zones (symbiosomes: II, *n* = 3; II‐III, *n* = 3; III, *n* = 3). j–l) Representative partial enlarged display of the e‐g. Scale bars = 0.3 µm. m), Quantification and comparison of the ER contact frequency and area of each symbiosome in different nodule zones (symbiosomes: II, *n* = 3; II‐III, *n* = 3; III, *n* = 3). All data are shown as mean ± standard deviation (SD). The statistical analysis was performed using the two‐way analysis of variance (ANOVA) and post‐hoc comparisons. The letters indicate values with statistically significant (*p* < 0.05) and non‐significant (*p* > 0.05) differences, respectively.

Next, we examined the morphological changes of ER along the nodule zones at different stages of symbiosome development. ^[^ ^25^ ^]^ As shown in **Figure** **1**a, the developing symbiosomes emerge randomly and entangle with ER tubules from the initial infection zone (zone II) to the transition zone (zone II‐III). In contrast, in the nitrogen‐fixing zone (zone III), ER tubules are fully expanded throughout the host cell and orient nearly parallel to mature symbiosomes that radiate regularly around a large central vacuole. Moreover, symbiosomes interact frequently with the ER through direct contact with each other throughout the symbiosome developmental stages (Figure 1a–c). In addition to the distribution, we also examined the morphological changes of ER by measuring the lumen width, and the width of ER surrounding the symbiosomes narrows gradually from zone II to zone III (Figure 1d). We also selected rhizobia‐infected cells with ultrathin high‐quality sections in each nodule zone (Movies S3–S5, Supporting Information) using the AutoCUTS‐SEM technique and generated 3D‐SEM images to show the reconstructions of symbiosomes and ER. It turns out that ER tubules with high curvature form cage‐like structures that arrest symbiosomes in zone II (Figure 1E; Movie S6, Supporting Information). In contrast, from zone II‐III (Figure 1f; Movie S7, Supporting Information) to zone III (Figure 1g; Movie S8, Supporting Information), symbiosomes are gradually enclosed by lamellar ER structures, characterized by connected flattening structures with lower curvature continuity on the Z‐axis of continuous slices. In addition, the volume and surface area of the ER surrounding symbiosomes increase with symbiosome enlargement from zone II to zone III (Figure 1h,i). Interestingly, the contact frequency between the ER and symbiosomes obviously increases alone with ER expansion (Figure 1j–m).

These subcellular features clearly demonstrate that the ER forms a cradle to support and coordinate the spatial distribution of symbiosomes in infected cells. Additionally, the ER exhibits a significant increase in surface area, volume, and contact sites between the ER and symbiosomes, with sustained morphological stretching and expansion of the lamella‐tubule‐lamella shift along with symbiosome accommodation and development. These results indicate that dynamic ER expansion plays a key role in symbiotic interface formation during symbiosome development.

### 2.2 Disturbance of ER Morphology Affects Symbiosome Development and Accommodation

**Figure 2:**
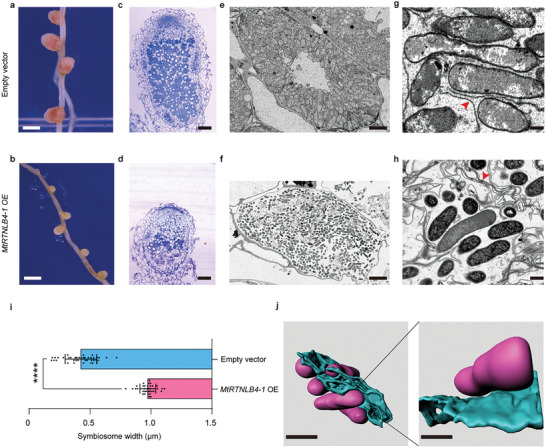
Disturbance of ER dynamics influences nodule development. Plasmids of *ProLjUb::MtRTNLB4‐1* and empty vector as control were introduced into *Medicago* A17 plants by hairy‐root transformation, respectively. GFP expressed from the construct was used as a selection marker for transformants and positive nodules were selected at 21days post‐inoculation (dpi). a–h) the nodule phenotype observation in the empty vector group (a, c, e, g) and the *MtRTNLB4‐1* overexpression group (b, d, f, h), including nodule growth (a, b) nodule resin section (c, d) SEM image of the mature infected cell (e, f), the symbiosome and ER distribution by local enlargement of E, F (g, h). The red arrowheads indicate ER. i) Quantification and comparison of symbiosomes width between the *MtRTNLB4‐1* overexpression group and the control group (*MtRTNLB4‐1* overexpression, *n* = 40; Empty vector, *n* = 40). All data are shown as mean ±SD. The statistical analysis was performed using the unpaired two‐sided Student's *t*‐test, ^****^ *p* < 0.0001. j) 3D reconstruction of the interaction and distribution of symbiosomes (magenta) and ER (blue) in nodule‐infected cells of *MtRTNLB4‐1* overexpression plants. Scale bars = 1 mm (a, b), 0.1 mm (c, d), 10 µm (e, f), 0.5 µm (g, h), 1 µm (left) and 0.8 µm (right) (j). At least two biological replicates were performed for each genotype.

To further validate that ER expansion is a prerequisite to symbiosome development, we took a genetic approach and ectopically expressed reticulons, which are called reticulon‐like proteins (RTNLs) in *M. truncatula*.^[^ ^26^ ^]^ RTNLs are membrane‐bending proteins that shape the ER membrane into tubules, and overexpression of RTNLs has been shown to prevent ER membrane expansion and sheet formation.^[^ ^27^, ^28^, ^29^ ^]^ We constructed constitutive expression vectors *ProLjUb*::*MtRTNLB4‐1* and *ProLjUb*::*MtRTNLB4‐2*, which were then introduced into *M.truncatula* ecotype A17 through hairy root transformation (Figure S3a,b, Supporting Information).^[^ ^30^ ^]^ We observed that both *MtRTNLB4‐1*‐ and *MtRTNLB4‐2*‐overexpressing nodules exhibit developmental decay with smaller sizes (**Figure** **2**a,b; Figure S3c–f, Supporting Information). Semi‐thin sections optical observation further revealed abnormally enlarged ITs and deficient nodule development, and staining of infected cells detect a low level of accommodation and developmental retardation of symbiosomes (Figure 2c,d). Subsequent SEM observation shows that the ER in infected cells generates longer unbranched tubules with local bundle aggregation (Figure 2e–h). Meanwhile, the symbiosomes around the unbranched ER tubules appear disordered, and their development is arrested (Figure 2e–i). Further 3D reconstruction results demonstrated that highly curved ER undergoes local aggregation without lamella expansion ^[^ ^20^, ^31^, ^32^, ^33^ ^]^ aggregation attenuates the interaction between the ER and symbiosomes (Figure 2j; Movies S9 and S10, Supporting Information). Taken together, these findings indicated that excessive ER tubulation impairs rhizobia accommodation and symbiosome development, further supporting the essential role of ER morphology transformation in symbiosis.

### 2.3 Stimulation of UPR Upon Rhizobia Infection and Colonization

The intensive increase in surface area and active morphology changes of the ER during symbiosis development reflect an effective regulation strategy behind it.^[^ ^18^ ^]^ Given the large protein synthesis requirements in the legume‐rhizobia symbiosis, we speculated that UPR plays a key role and active ER expansion meets the demand of robust UPR for successful symbiosis.^[^ ^20^, ^31^, ^32^, ^33^ ^]^

**Figure 3:**
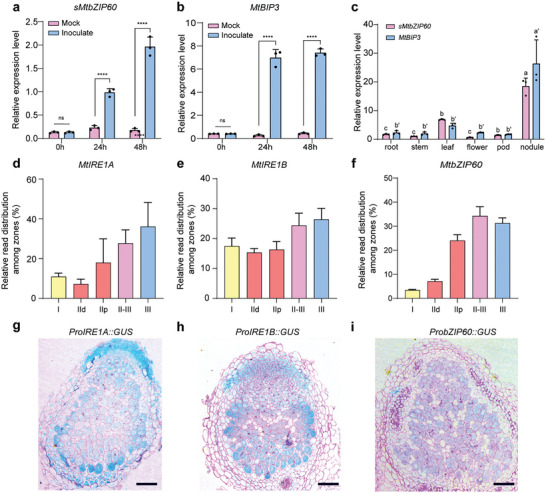
UPR induction during legume‐rhizobia symbiosis. a,b) Fluorescent quantitative PCR detection of spliced *MtbZIP60* (*sMtbZIP60*) (a) and *MtBIP3* mRNA (b) in early symbiosis (0, 24, 48h post‐inoculation, pi). c) Relative expression levels of *sMtbZIP60* and *MtBIP3* genes in multiple organs of the same plant. d–f) Relative expression levels of *MtIRE1A* (d), *MtIRE1B* (e), and *MtbZIP60* (f) along the symbiotic process are represented by distinct nodule sections. Zone I (meristematic), zone IId (distal) and zone IIp (proximal) constitute zone II (infection and differentiation zone). The data were obtained from the Symbimics website, curated from a previous publication,^[^ ^38^ ^],^ and represent the means of three technical replicates. Individual data points are not available. g‐i) Nodules at 14 dpi were dissected from *M. truncatula* transgenic lines expressing the β‐glucuronidase (GUS) reporter under the control of promoters of *MtIRE1A* (g), *MtIRE1B* (h) and *MtbZIP60* (i) respectively and stained with GUS solution for semi‐section. Ruthenium red staining was performed for imaging. Scale bars = 0.1 mm. For (a–c), all data are shown as mean ±SD. *n* = 3, ^****^ *p* < 0.0001 versus mock sample (a, b) and other organs (c). The statistical analysis was performed using the unpaired two‐sided Student's t‐test in (a, b) and ANOVA with post‐hoc comparisons in (c‐f). The letters indicate values with statistically significant (*p* < 0.05) and non‐significant (*p* > 0.05) differences, respectively.

To investigate the link between UPR and symbiosis, we first analyzed the expression profiles of two UPR marker genes.^[^ ^34^, ^35^, ^36^ ^]^ One is *bZIP60*, which is spliced by Inositol‐requiring enzyme type 1 (IRE1) into a spliced version of *bZIP60 (sbZIP60)* at the onset of ER stress, and the other is *BIP3*, which is one of the target genes of the sbZIP60 transcription factor and encodes a chaperone.^[^ ^37^, ^38^ ^]^ Quantitative real‐time PCR (qRT‐PCR) analyses showed that the abundance of *sbZIP60* mRNA and *BIP3* mRNA is significantly elevated compared to the control, indicating that rhizobia infection stimulates UPR in root cells (**Figure** **3**a,b). To investigate whether the enhanced expression of these two genes is relevant to the subsequent nodule development, we compared the expression levels of the two genes in different organs. As shown in Figure 3c, the expression of the two UPR marker genes is significantly higher in nodules than in the other organs, confirming the association of UPR with the legume‐rhizobia symbiosis.

We further examined the tissue‐specific expression pattern of three essential genes involved in UPR, including *MtIRE1A*, *MtIRE1B*, and *MtbZIP60* in various zones of the nodules (Figure 3d–f) using the dataset of a laser‐capture microdissection study together with RNA sequencing.^[^ ^39^ ^]^ We found that the transcripts of three genes tend to increase from zone II (IId, p) to zone III (ZIII) during the development and maturity of bacteroids for nitrogen fixation.

To further explore the expression patterns of the three genes in living cells, we detected the expression of the GUS reporter in nodule cells driven by the promoters of *MtIRE1A*, *MtIRE1B*, and *MtbZIP60* genes, respectively. The results show that expression of these three genes is specifically induced in infected cells (Figure 3g–i). Furthermore, we constructed *Pro35S*::*RFP‐MtbZIP60* to visualize its subcellular localization by fluorescence imaging of living cells, fluorescent signals were observed both at the ER and in the nucleus of infected cells (Figure S4d, Supporting Information), indicating that *MtbZIP60* mRNAs are spliced in infected cells and that the resulting truncated proteins are translocated into the nucleus.^[^ ^40^ ^]^ Collectively, these results verify the inherent UPR stimulation in the nodule cells from an intracellular perspective.

### 2.4 Symbiosome Development is Retarded in UPR‐Deficient Mutants

To assess the effect of UPR on the regulation of ER biogenesis in rhizobia‐infected cells and further symbiotic development, using CRISPR–Cas9‐mediated gene‐editing technology, we generated stable transgenic *Medicago* plants of *ire1a*‐, *ire1b*‐ and *bzip60*‐knockout mutants, respectively (Figure S5a–c, Supporting Information). Further phenotypic analysis revealed that, under non‐symbiotic conditions, those mutants do not show obvious growth defects compared to the wild‐type plants (Figure S5d–i, Supporting Information), consistent with the observations in previous *Arabidopsis* studies.^[^ ^41^ ^]^ We next evaluated their sensitivity to ER stress by treating the seedlings with tunicamycin (TM), which has been widely used to induce ER stress.^[^ ^42^ ^]^ Notably, upon TM treatment, the growth and development of the mutants are affected compared to the wild‐type plants, confirming that the knockout of these key players in UPR decreases the ability of mutants to deal with ER stress (Figure S6a–d, Supporting Information).

**Figure 4:**
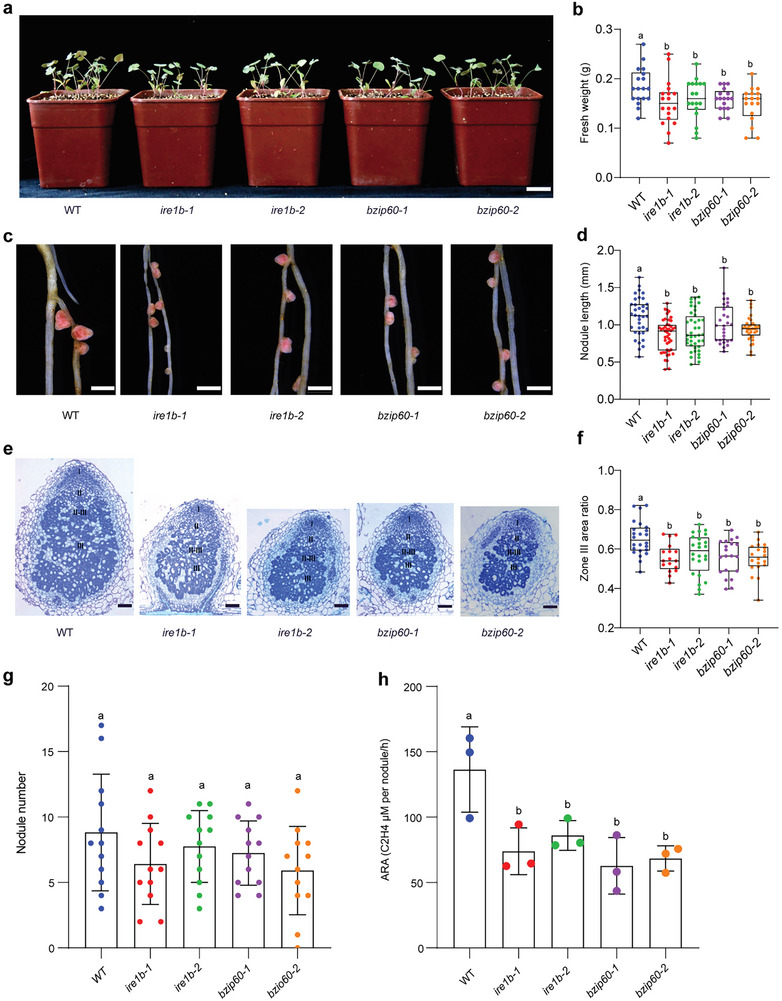
Deficiency in UPR function affects nodule symbiosis. a) Representative phenotypes in plant growth, from left to right: WT, *ire1b‐1*, *ire1b‐2*, *bzip60‐1*, *bzip60‐2*. b) Statistical analysis of the fresh weight of the above plants (WT *n* = 20, *ire1b‐1 n* = 20, *ire1b‐2 n* = 20, *bzip60‐1 n* = 19, *bzip60‐2 n* = 19). c) Representative phenotypes in nodules growth at 14 dpi, from left to right: WT, *ire1b‐1*, *ire1b‐2*, *bzip60‐1*, *bzip60‐2*. Scale bars = 2 mm. d) Quantitative statistical analysis on nodule length of the above plants (WT *n* = 36, *ire1b‐1 n* = 45, *ire1b‐2 n* = 40, *bzip60‐1 n* = 30, *bzip60‐2 n* = 33). e) Semi‐thin sections of nodules at 14 dpi were stained with 0.4% toluidine blue. Scale bars = 0.1 mm. f) Quantitative statistical results of area proportion in zone III. (WT, *n* = 24; *ire1b‐1*, *n* = 17; *ire1b‐2*, *n* = 24; *bzip60‐1*, *n* = 20; *bzip60‐2*, *n* = 20). g) Statistical result of pink nodules number at 14 dpi. *n* = 12. h) Acetylene reduction assay (ARA) revealed significantly decreased nitrogen‐fixing activities of *ire1b‐1*, *ire1b‐2*, *bzip60‐1*, and *bzip60‐2* nodules at 20 dpi. Each dot represents the mean value from 55 nodules (WT, *n* = 3; *ire1b‐1, n* = 3; *ire1b‐2, n* = 3; *bzip60‐1, n* = 3; *bzip60‐2, n* = 3). All data are shown as means ±SD. The statistical significance of the differences was tested using one‐way ANOVA and post‐hoc comparisons. Different and same letters indicate values with statistically significant (*p *< 0.05) and nonsignificant (*p* > 0.05) differences, respectively; The above nodules for semithin sections were collected from at least 60 plants with three biological replicates.

Importantly, these mutants provide valuable materials to investigate whether the UPR pathway regulates nodule symbiosis. We first analyzed early infection events, including the formation of infection foci, infection threads (ITs), and nodule primordia. Quantitative analysis showed that these UPR‐deficient mutants do not exhibit obvious defects during the early stages of infection (Figure S7a–f, Supporting Information). Next, we assessed nodule development in these UPR mutants and found that they formed smaller nodules compared to the wild type (**Figure** **4**a–d). The nodules of UPR mutants had a significantly reduced area proportion of the mature nitrogen fixation zone (zone III) relative to the wild type (Figure 4e–f). Consistently, although the nodule number in mutants is similar to that of the wild type (Figure 4g), the nitrogenase activity in the mutant nodules is dramatically lower than in the wild type with an equal number (Figure 4h). Collectively, these results indicate that the development of symbiosomes is retarded in the UPR‐deficient mutants.

It is worth mentioning that the *ire1a* mutant does not exhibit clear symbiotic development defects comparable to *ire1b* and *bzip60* (Figure S8a–g, Supporting Information). Compared with the results of the nodule single‐cell sequencing database, the expression level of the *IRE1A* gene is significantly lower than that of the *IRE1B* gene (Figure S8h, Supporting Information).^[^ ^43^ ^]^ This is further confirmed by the expression analysis by using different tissues of *M. truncatula* (Figure S8i, Supporting Information). Thus, *IRE1B* may play a more critical role in UPR regulation of the symbiotic process than *IRE1A*.

### 2.5 ER Expansion Couples with Enhanced UPR to Achieve Rhizobia Accommodation and Symbiosome Development

**Figure 5:**
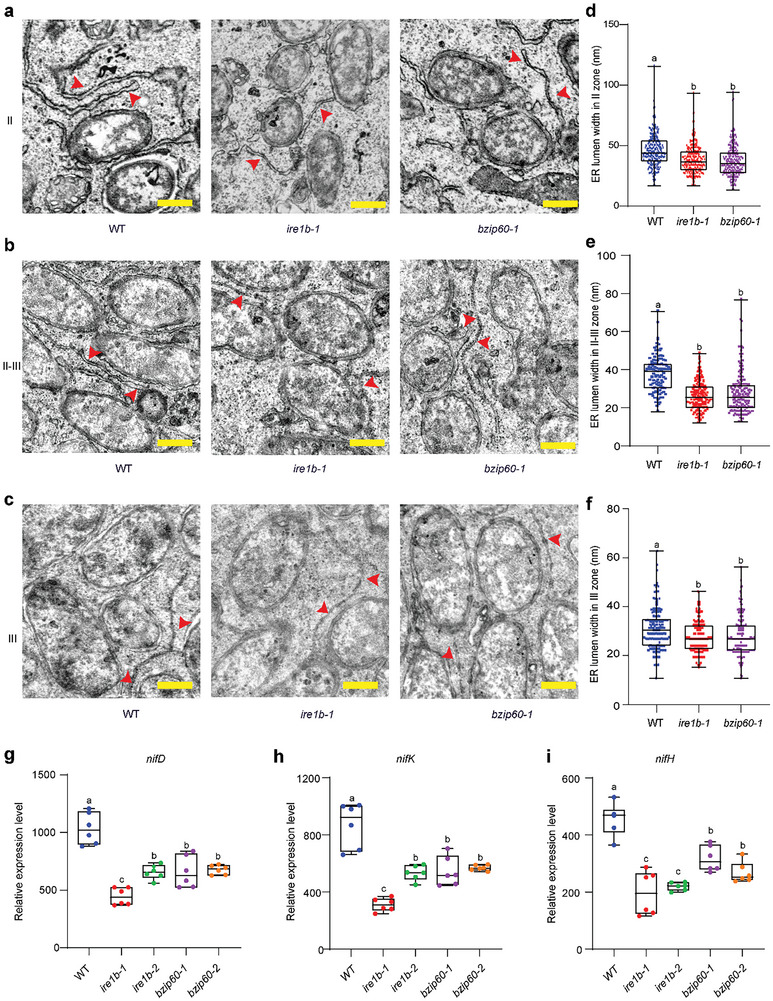
Alteration of ER biosynthesis in UPR mutants results in defects in nitrogen fixation. a–c) Representative SEM images of 2‐week‐old nodules in zone II (a), zone II‐III (b), and zone III (c), from left to right: WT, *ire1b‐1*, *bzip60‐1*. The ER lumen was indicated by the red arrow. Scale bars = 0.5 µm. d) Quantitative analysis of the ER lumen width in zone II. (WT, *n* = 300; *ire1b‐1*, *n* = 293; *bzip60‐1*, *n* = 297). e) Quantitative analysis of the ER lumen width in zone II‐III. (WT, *n* = 118; *ire1b1*, *n* = 183; *bzip60‐1*, *n* = 170). f) Quantitative analysis of the ER lumen width in zone III. (WT, *n* = 183; *ire1b‐1*, *n* = 185; *bzip60‐1*, *n* = 183). All data are shown as mean ±SD. Statistical analysis was performed with the one‐way ANOVA method with post‐hoc comparisons. The above SEM statistical results were randomly selected from 3 cells in each zone. g‐i) Transcript level of *nifD* (g), *nifK* (h), *nifH* (i) was determined by qRT‐PCR, and the rhizobia housekeeping gene *rpoA* was used for normalization, *n* = 6. All data are shown as mean ±SD. The statistical analysis was performed by the two‐way ANOVA and post‐hoc comparisons. The letters indicate values with statistically significant (*p* < 0.05) and non‐significant (*p* > 0.05) differences, respectively.

Effective UPR alleviates ER stress by enhancing ER membrane expansion to provide more ER surface and luminal space. To investigate whether this holds true in our case, we examined the ER alterations in the two UPR‐deficient mutants to determine if the ER membrane expansion of infected cells is also affected. Notably, the ER lumen width of infected cells narrows significantly within the entire three zones (as mentioned earlier) in UPR mutant nodules, such as *ire1b‐1* and *bzip60‐1*, compared to the WT (**Figure** **5**a–f). These results indicate that functional deficiency of UPR leads to reduced ER membrane expansion and defective symbiosome development, and UPR is indeed involved in the regulation of ER morphology and symbiosome accommodation during symbiotic development.

To further validate the importance of ER morphology changes resulting from enhanced host UPR for symbiosomes development, we first examined the differentiation of bacteroid and found no severe developmental defects, such as early bacteroid senescence and degradation (Figures 4e and 5a–c). We next analyzed the expression levels of *nifD*, *nifK*, and *nifH*, which encode nitrogenases of N_2_ fixation in rhizobia.^[^ ^44^ ^]^ The qPCR results show that the expression levels of *nifD*, *nifK*, and *nifH* decrease significantly in the above mutants compared to the wild type (Figure 5g–i). Together, these results confirm that UPR functional deficiency impairs nodule development and nitrogen fixation.

Since *RTNLB4‐1* overexpression results in abnormal expansion of the ER membrane (see Figure 2j), we asked whether such a phenotype is associated with UPR deficiency. Indeed, the expression of both the two UPR markers (*MtbZIP60* and *MtBIP3*) and the downstream *CALNEXIN* genes (*MtCNX1* and *MtCNX2*) and *PROTEIN DISULFIDE ISOMERASE* gene (*MtPDI*)^[^ ^45^ ^]^ appeared to be down‐regulated in the transgenic plants (Figure S9, Supporting Information). Collectively, these results indicate an immediately reciprocal feedback regulation between the abnormal ER membrane expansion and the weakened UPR signaling.

**Figure 6:**
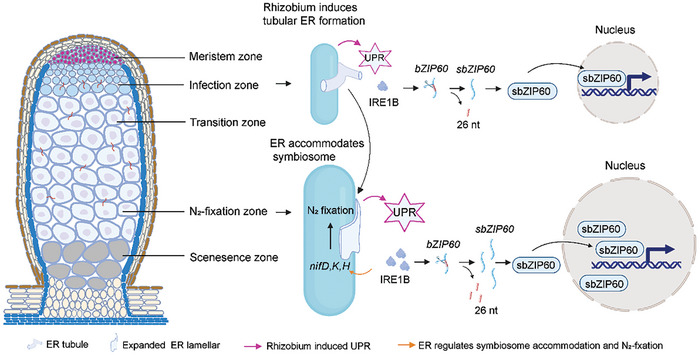
Dynamic ER expansion couples with enhanced UPR to achieve rhizobia endosymbiosis. The schematic diagram illustrates the model of ER expansion coupled with UPR during root nodule symbiosis. The host ER surrounding symbiosomes undergoes dynamic expansion from tubules to lamellae to support the accommodation and development of symbiosomes, which in turn induces host UPR activation. During UPR, IRE1B splices the mRNA of *bZIP60*, and the resulting sbZIP60 protein translocates into the nucleus to activate downstream genes to further regulate ER expansion surrounding symbiosomes and consequent nitrogen fixation. The figure was created with BioRender software.

Taken together, we propose that the host ER surrounding the symbiosomes undergoes dynamic expansion, and the compartmentalization of the ER controls the rhizobia intracellular colonization. Importantly, ER expansion couples with enhanced UPR to achieve rhizobia accommodation and symbiosome development during the legume‐rhizobia symbiosis. Thus, function deficiency of UPR in *ire1b* and *bzip60* mutants causes defective ER structures and impaired symbiosome development (**Figure** **6**).

## 3 Discussion

Symbiosis is a reciprocally beneficial process for the plant and bacterial partners. Understanding the cellular and molecular mechanisms involved in the accommodation of symbiosomes is a huge challenge to study the endosymbiosis process during legume symbiosis. Our study unveiled two important features of host ER‐related functions in the process of symbiosis. One aspect is the dynamic expansion and remodeling of the host ER along with the symbiosome accommodation, and the other is the stimulation of UPR in the infected cells, which is required to shape nodule symbiosis throughout the stages of symbiosome development.

Cells constantly adjust the sizes and shapes of their organelles according to physiological requirements.^[^ ^46^ ^]^ It has been well documented that the ER can connect directly with other organelles and undergo size and shape changes in the complicated membrane network to regulate different cellular activities in eukaryotic cells.^[^ ^14^, ^47^, ^48^ ^]^ In our study, we observed that the ER changes its size and shape spatiotemporally along with the development of symbiosis. At different stages of symbiosome development, ER increases its surface area and volume coordinately with the expanding of symbiosomes; in multiple zones of nodule, the dynamic arrangement of the ER is well matched with the size enlargement of symbiosomes; and ER morphology is transformed from tubules to lamellae with the membrane expansion to adapt the growth and development of symbiosomes in the infected cells. Consistently, the disturbance of ER morphology through *RTNLBs* overexpression leads to disorganization and defects in symbiosome development. Thus, our results provide a solid line of evidence showing the dynamic changes of ER surface area and the morphology patterns in coupled with the process of symbiosome accommodation and development. Thus, our results provide a solid line of evidence showing that the dynamic changes of ER surface area and the changes in patterns of morphology are coupled with the process of symbiosome accommodation and development.

It has been reported that perturbation of ER homeostasis causes ER stress, which, in turn, activates UPR and enhances protein and lipid synthesis to expand ER membranes and volume in yeast and mammalian cells.^[^ ^31^, ^49^, ^50^ ^]^ Based on the observation of enhanced biosynthesis of ER surface and volume along with the process of symbiosis, we predicted that the intense production of membrane components could result in unfolded protein accumulation, and the ER processing capacity might become insufficient for the requirements of protein folding under this circumstance, thus leading to the occurrence of ER stress. As a result, UPR and related reprogramming events were triggered to alleviate ER stress and maintain ER homeostasis. Indeed, through cellular, molecular, and genetic analyses, we obtained convincing results showing that high‐intensity execution of UPR exists in nodules, where rhizobia undergo intracellular accommodation and colonization. Consistently, in mutants with knock out of UPR‐related genes such as *ire1b* and *bzip60*, ER membrane biogenesis capacity decreased significantly compared to the wild type, associated with the formation of smaller mature zone III, which established a communication hub between the necessity of the ER membrane biogenesis mediated by UPR and nodule development. In addition, we tested the effect of UPR at the late stage of symbiosis by examining the decreased expression level of *nifD, nifK*, and *nifH* genes in the nodules of relevant mutants, which further indicated that UPR could mediate balance beneficial for the survival and function persistence of bacteroids. Taken together, these data demonstrated that maintaining dynamic morphological transformation of the ER is a requirement for nodule development. By means of ER membrane expansion, larger space could be supplied to hold more misfolded proteins. Moreover, there likely exist reciprocal feedback regulations between the ER membrane expansion and the UPR signaling. In other words, UPR plays positive regulatory roles in symbiosome and nodule development.

Our findings uncovered ER expansion and UPR induction as two key events in symbiosome development in *M. truncatula nodules*, and further revealed the intrinsic coupling of ER morphology with proper UPR function during root nodule symbiosis by IRLC legumes. It should be noted that, anatomically, at least two distinct forms of intracellular accommodation of symbiotic bacteria in nitrogen‐fixing legume nodules have been identified. One type, known as SYM‐type nodules, which involves rhizobia being released from infection threads and enclosed within symbiosome membrane, is observed in most genera of the legume subfamily Papilionoideae, such as *Pisum* and *Medicago*, in all nodulated genera of the tribe Mimosae in the legume subfamily Caesalpinioideae, as well as in non‐arboreal species of the Caesalpinioid genus *Chamaecrista*.^[^ ^51^, ^52^ ^]^ The other type, known as FT‐type nodules, in which rhizobia are retained within specialized, thin‐walled infection threads called fixation threads (FTs), are found in a few early‐branching Papilionoid legumes, most nodulated non‐Mimosoid Caesalpinioid legumes (the exception being arboreal *Chamaecrista* spp.),^[^ ^52^ ^]^ and in nodules on *Parasponia* (Cannabaceae), the only known non‐legume capable of forming nodules^[^ ^1^ ^]^. In the present study, we demonstrated that ER dynamic expansion and UPR activation are essential for symbiosome accommodation and development in *M. truncatula*, which has SYM‐type nodules. Interestingly, however, recent TEM observations have shown that FTs in FT‐type nodules on Caesalpinioid nodules are also surrounded by a cell membrane arising from ER, thus it is intriguing to investigate the interplay between ER remodeling and UPR in a wider set of symbiotic nodule types in future studies.^[^ ^51^ ^]^ A deeper understanding of the cellular basis on the root nodule symbiosis would unlock new strategies for optimizing nitrogen fixation in crops and contribute to sustainable farming practices in the future.

## 4 Experimental Section

### Biological Materials and Growth Conditions

*M. truncatula* ecotypes A17 and R108 were used in this study. *M. truncatula* seeds were scarified with sulfuric acid (SCR, 7664‐93‐9, China) for 10 min, rinsed with sterile water fifteen times, and surface‐sterilized in 6% sodium hypochlorite (SCR, 7681‐52‐9, China) for 5 min, and then washed fifteen times with sterile water. Sterilized seeds were stratified on inverted agar plates for 24 h at 4 °C and germinated overnight at 22 °C before being transferred to sterile perlite pots or used for hairy‐root transformation. Plants were grown in a greenhouse under controlled conditions with a 16‐h light/8‐h dark photoperiod and a constant temperature of 22 °C. They were watered every four days. For inoculation, the *Sinorhizobium meliloti* strain 2011 (Sm2011) was used. Hairy‐root transformation was performed using *Agrobacterium rhizogenes* strain MSU440, following a previously described protocol.^[^ ^53^ ^]^

### Scan Electronic Microscopy

Nodule samples were fixed with 2.5% (vol/vol) glutaraldehyde with 0.1 M phosphate buffer (PB, Na_2_HPO_4_^.^12H_2_O, NaH_2_PO_4_^.^2H_2_O) (pH 7.4), and then washed four times in PB. Then they were first immersed in 1% (wt/vol) OsO_4_ (Ted Pella, 20816‐12‐0, USA) and 1.5% (wt/vol) K_3_[Fe (CN)_6_] (Sigma‐Aldrich, 13746‐66‐2, USA) aqueous solution at 4 °C for 1 h. After washing, nodules were incubated in filtered 1% thiocarbohydrazide aqueous solution (Sigma‐Aldrich, 2231‐57‐4, USA) at room temperature for 30 min, 1% unbuffered OsO_4_ aqueous solution at 4 °C for 1h and 1% Uranyl acetate aqueous solution (ZXBR, 6159‐44‐0, China) at 4 °C overnight following four rinses in double distilled water for 10 min each between each step. Then tissues were dehydrated through graded alcohol (30, 50, 70, 80, 90, 100, and 100%, 10 min each at 4 °C) into pure acetone (Sigma–Aldrich, 67‐64‐1, USA). Samples were infiltrated in a graded mixtures (3:1, 1:1, 1:3) of acetone and SPI‐PON812 resin (SPI Supplies, 905929‐77‐4, USA) (including 16.2 mL SPI‐PON812 monomer, 10 mL dodeceny succinicanhydride (DDSA), and 8.9 mL N‐Methylol acrylamide (NMA), 1.5% N‐dimthylbenzylamine (BDMA), then changed pure resin. Finally, tissues were embedded in pure resin with 1.5% BDMA and polymerized for 12 h at 45 °C, 48 h at 60 °C.

### AutoCUTS‐SEM and 3D Reconstruction

Automatic collector of ultrathin sections scanning electron microscopy (AutoCUTS‐SEM) was performed as previously described.^[^ ^54^ ^]^ For the Large‐scale 3D reconstruction study, 200 sections were collected by the ultramicrotome (Leica, UC7, Germany) with the AutoCUTS device for each sample. Next, high throughput serial sections were finally automatically acquired by Scanning Electron Microscope (FEI, Helios Nanolab 600i dual‐beam SEM, USA) with automated software (AutoSEE), and an image reconstruction program was conducted. The image parameters include an accelerating voltage of 2 kV, beam current of 0.69 nA, CBS detector, pixel size of 58.6 nm, and dwell time of 5 µs. For 3D reconstruction, manual segmentation of cells and their associated processes were performed using commercial software Imaris (Bitplane, version9.0, Switzerland). The stack of labeled images was exported and processed further for 3D rendering and data analysis.

### Phylogenetic Analysis

The 19 protein sequences of 15 *MtRTNLBs* gene family were obtained from Phytozome13 (http://phytozome.jgi.doe.gov/pz/portal.html) and protein sequences were aligned using ClustalW, and the phylogenetic tree was carried out using the neighbor‐joining method of MEGA11, the bootstrapping value was set at 1000 replications to evaluate the consistency of the analysis. The motifs analysis was accomplished by the MEME server (http://meme‐suite.org/index.html). The TB tools were used to visualize combined results.^[^ ^30^ ^]^

### Plasmid Construction

As described in the previous study, the 2 × Phanta Max Master Mix DNA polymerase with high‐fidelity (Vazyme, P515‐01, China) was used to amplify the full‐length cDNA of *MtRTNLB4‐1*, *MtRTNLB4‐2, MtbZIP60* and ≈2000‐bp upstream gDNA (promoter) sequence of *MtIRE1A* (Medtr8g073190), *MtIRE1B* (Medtr5g024510), *MtbZIP60* (Medtr1g050502) from *M. truncatula* A17. To further obtain the *pCambia1391‐ProIRE1A/IRE1B/bZIP60‐GUS* constructs, ClonExpress II One Step Cloning Kit (Vazyme, 7E771E3, China) was used to ligate the *ProIRE1A/IRE1B/bZIP60* fragment, and *Pst* I‐ (NEB, R0140V, USA) and *Bam*H I‐ (NEB, R0136V, USA) linearized *pCambia1391* binary vector. For the constitutive overexpression constructs *ProLjUb::MtRTNLB4‐1*, *ProLjUb::MtRTNLB4‐2*, ClonExpress II One Step Cloning Kit to ligate target fragments and *Kpn* I HF‐ (NEB, R3142S, USA) and *Xba* I‐(NEB, R0145L, USA) linearized *ProLjUb‐Pro35S::GFP* binary vector was used. For the One Step Constructs were confirmed by Sanger sequencing. (Table S1, Supporting Information).

### RNA Extraction and qPCR

Total RNA was extracted as the previously described method.^[^ ^55^ ^]^ Briefly, ground sample powder was homogenized in TRIzol reagent (Thermo Fisher, 380 511, USA) by vortexing at high speed for 1 min at room temperature. After a 5 min incubation, chloroform was added to the mixture, followed by vortexing for 1 min and centrifugation at 12000 rpm for 10 min to separate the phases. The upper aqueous phase was carefully transferred to a fresh RNase‐free tube and mixed with an equal volume of ice‐cold isopropanol. The mixture was centrifuged at 12000 rpm for 10 min at 4 °C to precipitate the RNA. The resulting pellet was washed with 200 µL of 70% ethanol and centrifuged at 7000 × g for 5 min. The supernatant was decanted without disturbing the pellet, and the RNA pellet was air‐dried. Finally, the RNA was resuspended in 30 µL of RNase‐free deionized water. 2 µL of total RNA for each sample was applied for reverse transcription using the SuperScript III First‐Strand Synthesis System (Vazyme, 7E731J3, China) with the mixture of oligo (dT) primers and random primers. The RT‐qPCR assays were performed using the SYBR Green real‐time PCR master mix (Vazyme, 7E0813G4, China) with a Real‐time fluorescence quantitative PCR system (BioRad, CFX96, USA). The expression of plant genes and bacterial genes were normalized to *MtACTIN11* or *rpoA* expression, respectively. All of the reactions were performed three times independently. A list of the primers used for RT‐qPCR is provided in Table S1 (Supporting Information).

### Histochemical Staining, Resin Embedding and Sectioning

The promoter regions upstream of the start codon (ATG) of *MtIRE1A* (2262 bp), *MtIRE1B* (2029 bp), and *MtbZIP60* (2474 bp) were amplified from *M. truncatula* A17 genomic DNA. These promoter sequences were then cloned into the *pCAMBIA1391z* vector. The resulting constructs were introduced into *Agrobacterium tumefaciens* strain EHA105, which was subsequently used to transform *M. truncatula* R108 via stable transformation. Transgenic seedlings were screened by PCR amplification and sequencing using GUS‐specific primers (Table S1, Supporting Information). Transformed plants were inoculated with *Sinorhizobium meliloti* strain Sm2011. At 14 dpi, nodules were harvested and immersed in 5‐Bromo‐4‐chloro‐3‐indoxyl‐beta‐D‐glucuronide (X‐Gluc) (Lablead, 114162‐64‐0, China) staining buffer for 8 h to detect GUS activity. For detailed analysis, 10‐µm nodule sections were prepared using a Leica RM2265 microtome, stained with 0.1% Ruthenium Red (Sigma–Aldrich, 11103‐72‐3, USA), and visualized using a Leica M205FA microscope equipped with a DFC450c camera. Additionally, 5‐µm nodule sections were stained with 0.05% toluidine blue (Amresco, 672–5, USA) for nodule structure visualization.

### Subcellular Localization and Confocal Microscopy

Live‐cell imaging was performed using a spinning‐disk confocal microscope (PerkinElmer, UltraView VoX, USA) equipped with a Yokogawa Nipkow CSU‐X1 spinning‐disk scanner, Hamamatsu EMCCD 9100–13, Nikon TiE inverted microscope with the Perfect Focus System45. For fluorescence imaging, the *Pro35S* *:*:*HDEL‐RFP* and *Pro35S* *:*:*RFP‐MtbZIP60* vectors were transformed into *M. truncatula* A17 by *Agrobacterium rhizogenes* transformation. The transformed plants were infected with Rhizobium Sm2011. 21‐day‐post‐inoculation nodules were hand‐sectioned using a double‐edged razor blade and immediately immersed at 2 µg mL^−1^ solution of 4′,6‐diamidino‐2‐phenylindole (DAPI) (Roche, 236 276, Switzerland) for 5 min. To visualize the interaction between symbiosomes and the ER in nodule cells, hand‐sectioned nodules expressing HDEL‐RFP were submerged in SYTO9 (Thermo Fisher, S34854, USA), stained for 5 min, and briefly washed with distilled water. Acquired images were processed and analyzed using the software of Volocity (Perkin Elmer, version 6.3, USA) and Image J software as described previously.^[^ ^55^ ^]^

### CRISPR–Cas9‐Mediated Gene Editing

CRISPR–Cas9‐mediated gene editing was performed as described previously.^[^ ^55^, ^56^ ^]^ gRNA sequences were used to guide Cas9 to the exons of *MtIRE1A, MtIRE1B, MtbZIP60* (Table S1, Supporting Information). gRNA was cloned into the *pAtU6‐26‐sgRNA‐SK* vector. The cassette containing the sgRNA was then cloned into the *pCAMBIA1300‐ProAtUBQ10::Cas9* binary vector and used to transform *Agrobacterium* EHA105, which was then transformed into *M. truncatula* R108. The mutants were screened by sequencing using gene‐specific primers (Table S1, Supporting Information).

### UPR‐Defective Mutant Phenotyping

For normal growth analysis, plants were grown in vermiculite supplemented with FM medium and sampled at 12 and 20 days for phenotypic evaluation. At least 16 plants per genotype were analyzed. For infection event analysis, five‐day‐old seedlings of *Medicago* wild‐type R108 and UPR‐defective mutants, grown in vermiculite with FM medium, were inoculated with Sm2011‐GFP (OD_600_ = 0.02). Plants were harvested at 3, 5, and 7 dpi to examine infection events using a Nikon confocal microscope equipped with photomultiplier tubes (PMTs). At least 8 plants from each genotype were used for quantification of infection events at each time point. For nodulation phenotype analysis, nodules from wild‐type (R108) and UPR‐defective mutants were harvested at 21 dpi. At least 36 plants per genotype were used for this analysis.

### Acetylene Reduction Assay

The *M. truncatula* seedlings were grown on vermiculite and inoculated with rhizobium Sm2011. Nodules at 21 dpi were collected and put into a closed 20 mL vial containing 2 mL acetylene (C_2_H_2_) at 28 °C for 3 h. For each sample, three biological replicates were performed for analysis. Acetylene was measured using a gas chromatograph (EWAI, GC‐4000A, China).

### ER Stress Treatment Assay

For ER stress assay, 5‐day‐old seedlings were transferred to FM culture medium containing 0.5 µg mL^−1^ TM (Aladdin, 11089‐65‐9, China) with DMSO as the solvent or DMSO‐only as mock, 20 seedlings per replicate. Then, the seedlings grew vertically under the normal growth condition. After 7 days, the seedlings primary root length was measured. The length of primary roots was photographed and further measured using ImageJ software. The above experiments were performed three times independently with consistent results.

### Bioinformation Prediction

The UNAFold Web Server(http://www.unafold.org/mfold)was used to predict the RNA secondary structure of *MtbZIP60*. The full structure information was shown in the source data (Figure S4, Supporting Information). The cNLS Mapper Web Server (https://nls‐mapper.iab.keio.ac.jp/cgi‐bin/NLS_Mapper_form.cgi) was used to predict the nuclear localization signal. The TMHMM (http://www.cbs.dtu.dk/services/) TMHMM/Web Server was used to predict the transmembrane domain of MtbZIP60.

### Statistical Analysis

Statistical analysis was performed using GraphPad Prism (version 8.0.2). Results were expressed as means ± standard deviation (SD). To determine statistical significance, an unpaired two‐sided Student's t‐test was used for comparisons between the two groups, statistical significance was denoted as follows: ^*^ *p*<0.05, ^**^ *p*<0.01, ^***^ *p*<0.001, ^****^ *p*<0.0001, and ns indicates not significant. Then multiple *t*‐tests and one‐way ANOVA were employed for comparisons involving multiple groups. Different and same letters indicate values with statistically significant (*p* < 0.05) and nonsignificant (*p* > 0.05) differences, respectively.

## Conflict of Interest

The authors declare no conflict of interest.

## Author Contributions

J.R. and Q.W. contributed equally to this work. J.R. and Q.W. designed the research plan and experiments. J.R. performed experiments, prepared figures and videos, and wrote the original draft. Q.W. performed experimental observation and interpreted the data and revised the manuscript. X.Z., Y.C., J.W., Y.Y., and J.T. provided essential technical assistance. G.Q. reviewed and edited the manuscript. Z.K. conceived the project, interpreted the data, wrote and revised the article.

## Supporting information



Supporting Information

Supplemental Movie 1

Supplemental Movie 2

Supplemental Movie 3

Supplemental Movie 4

Supplemental Movie 5

Supplemental Movie 6

Supplemental Movie 7

Supplemental Movie 8

Supplemental Movie 9

Supplemental Movie 10

## Data Availability

The data that support the findings of this study are available from the corresponding author upon reasonable request.
