# Supplementary material for: Control of Rhizobia Endosymbiosis by Coupling ER Expansion with Enhanced UPR
Source: Adv Sci (Weinh). 2025 Feb 22;12(15):2414519. doi: 10.1002/advs.202414519 (PMC12005732; doi:10.1002/advs.202414519)
Supplement: Supplementary file 1 — Supporting Information [file ADVS-12-2414519-s008.docx]

Supporting Information

Control of Rhizobia Endosymbiosis by Coupling ER Expansion with Enhanced UPR

*Jing Ren, Qi Wang, Xiaxia Zhang, Yongheng Cao, JingXia Wu, Juan Tian, Yanjun Yu, Qingqiu Gong, Zhaosheng Kong ^*^*

J. Ren, Q. Wang, X. Zhang, Y. Cao, J. Wu, J. Tian, Y. Yu, Z. Kong

State Key Laboratory of Plant Genomics

Institute of Microbiology, Chinese Academy of Sciences

Beijing 100101, China

E-mail: [zskong@im.ac.cn](mailto:zskong@im.ac.cn)

J. Ren, Y. Cao, J. Wu, Z. Kong

University of Chinese Academy of Sciences

Beijing 100049, China

Q. Wang, Z. Kong

Houji Laboratory in Shanxi Province, Academy of Agronomy

Shanxi Agricultural University

Taiyuan 030031, China

Q.Wang

Department of Plant Microbe Interactions

Max Planck Institute for Plant Breeding Research

Cologne 50829, Germany

Q. Gong

State Key Laboratory of Microbial Metabolism & Joint International Research Laboratory of Metabolic and Developmental Sciences, School of Life Sciences and Biotechnology

Shanghai Jiao Tong University

Shanghai 200240, China


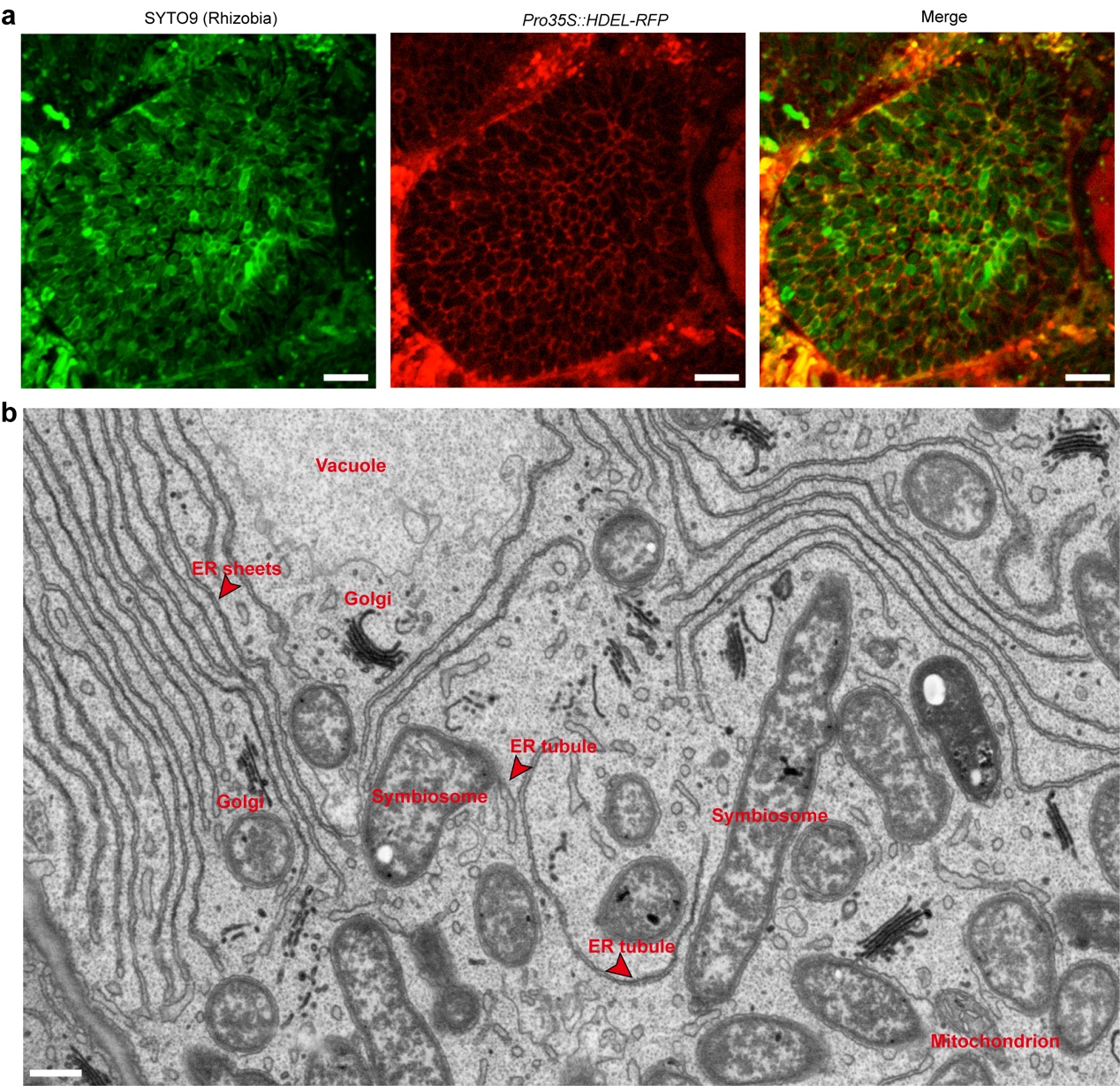


**Figure S1**. Developing symbiosomes interact with organelles in the infection zone. a) Two-color overlay images showing symbiosomes residing within honeycomb-like ER structures (labeled with HDEL-RFP, an ER marker) in *Pro35S::HDEL-RFP*-expressing nodules. Hand-sectioned samples of three-week-old nodules expressing *Pro35S::HDEL-RFP* were used to analyze the subcellular distribution of the ER. *Sinorhizobium meliloti* strain Sm2011 cells were stained with SYTO9. Scale bars = 12 μm. At least 60 rhizobia-infected cells from 15 nodules were analyzed across three independent experiments. b) A wide-view SEM image of developing symbiosomes in zone II of M. truncatula nodules, showing frequent interconnections with the ER, Golgi apparatus, and mitochondria. At least 10 cells were analyzed, with consistent results observed. Scale bar = 0.5 µm.


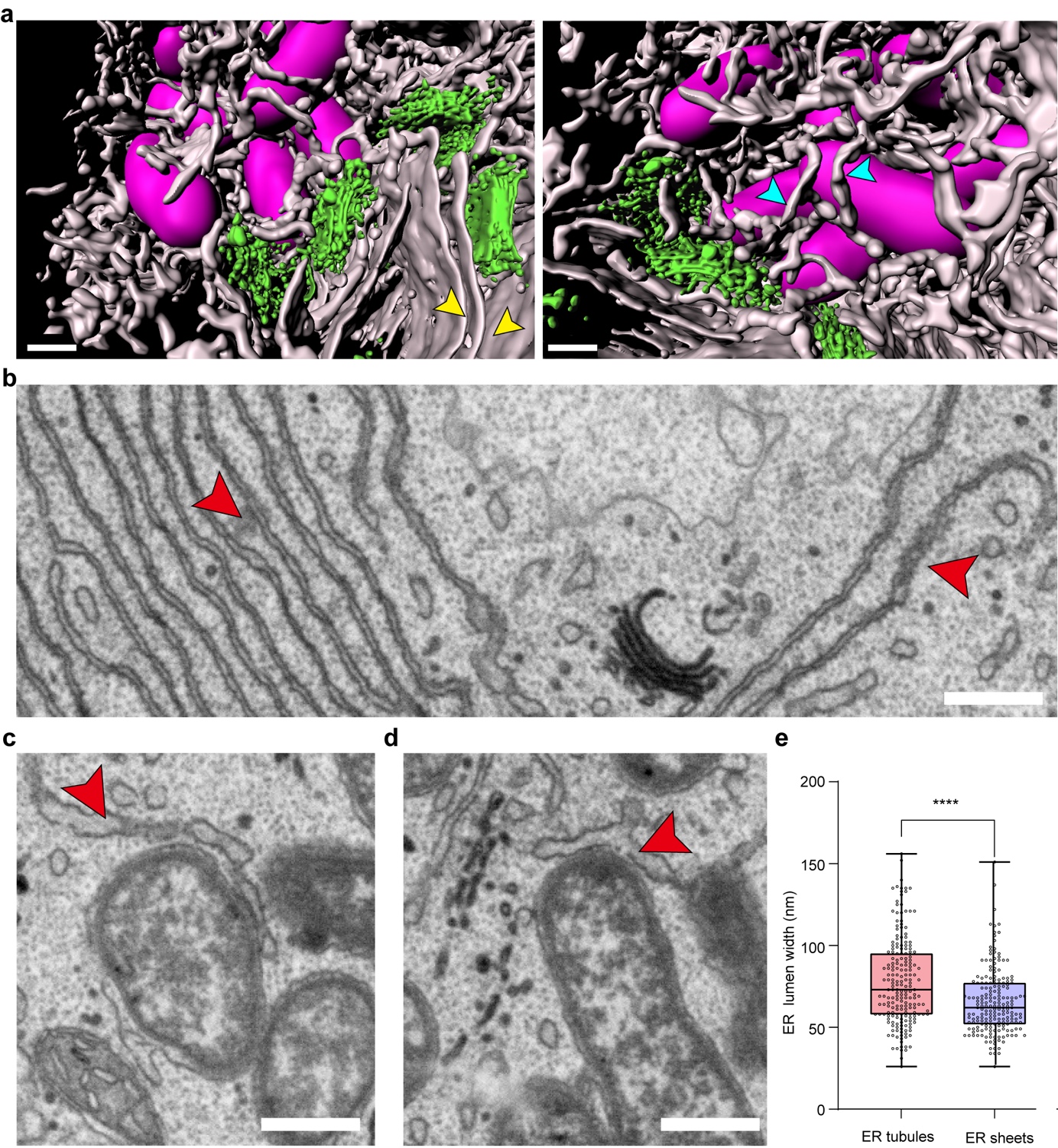


**Figure S2**. ER morphology transformation from lamellar sheets to tubules in infection zone. a) 3D reconstructions of symbiosomes, ER, and Golgi apparatus in the infection zone (zone II) of 3-week-old nodules. The ER transforms from lamellar sheets into tubular structures to capture rhizobia. Yellow arrowheads indicate ER sheets, and blue arrowheads indicate ER tubules. ER, Golgi apparatus, and symbiosomes are colored gray, green, and magenta, respectively. Scale bar = 0.5 µm. b-d) Local views of SEM images showing ER sheets (b) and ER tubules (c, d). Scale bars = 0.5 µm. e) Statistical comparison of ER lumen width between ER sheets and ER tubules. Statistical analysis was performed using an unpaired Student’s t-test; *****p* < 0.0001 (ER sheets *n* = 200, ER tubules *n* = 200). Two cells from the infection zone were reconstructed, showing consistent results.


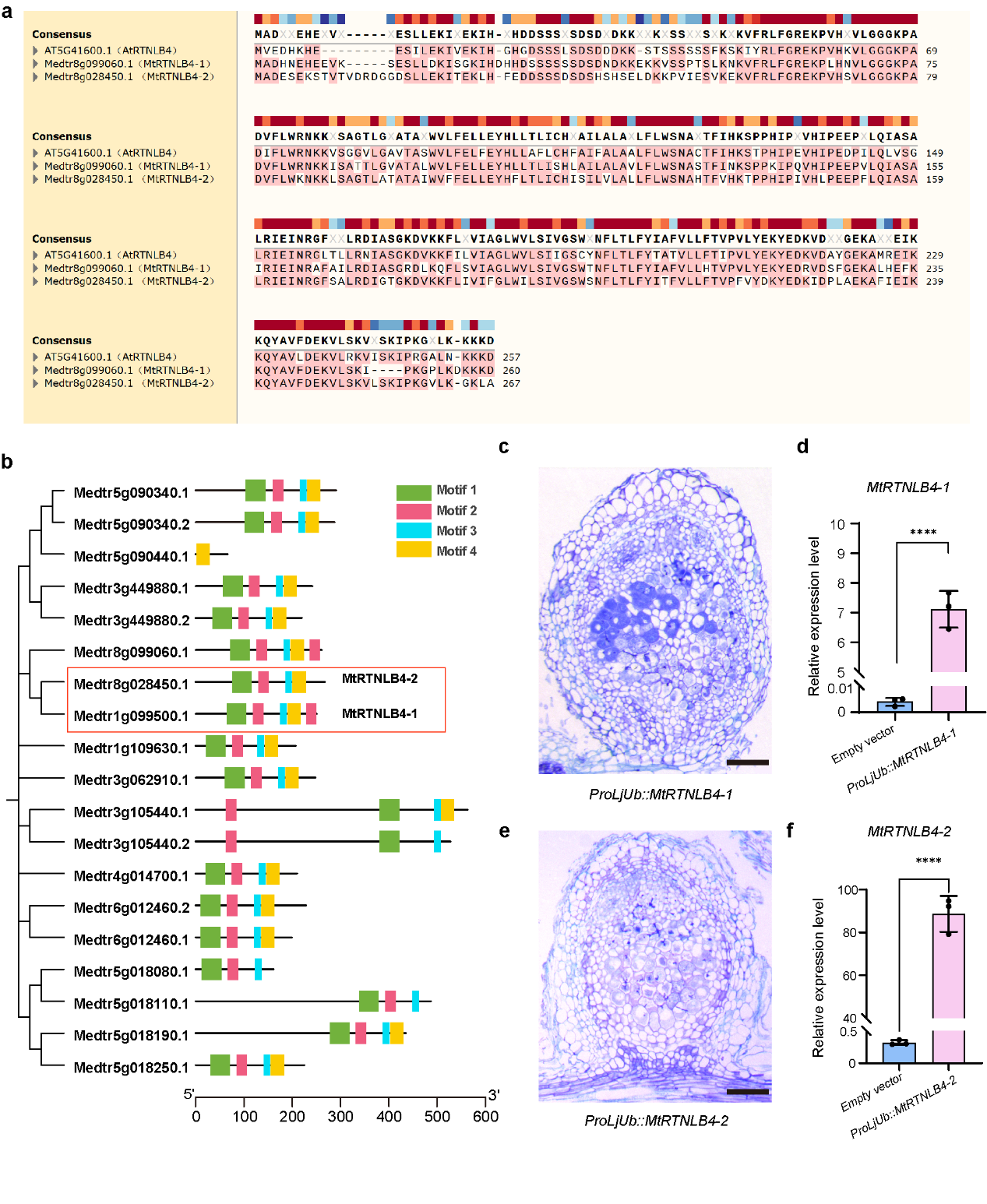


**Figure S3.** Sequence homology and phylogenetic analysis of MtRTNLB protein family. a) Protein sequences alignment of *AtRTNLB4* and two *MtRTNLBs* with highest homology. The alignment was performed using Muscle method. b) Phylogenetic relationships of *MtRTNLBs*. The evolutionary history was inferred by using the Maximum Likelihood method. The graphical diagrams display the motif distribution in the respective proteins. The scale indicates the length of amino acid of proteins. c-f) Representative images of semi-thin nodule sections transformed with *ProLjUb::MtRTNLB4-1* (c) and *ProLjUb::MtRTNLB4-2* (e). Scale bars = 0.1 mm. The transcript level of *MtRTNLB4-1* (d) and *MtRTNLB4-2* (f) was determined by qRT-PCR and the housekeeping gene *MtACTIN11* was used for data normalization. Bars and error bars indicate the mean and the standard deviation, respectively. Statistical analysis was performed with unpaired Student’s t-test, *****p* < 0.0001, (*n* = 3).


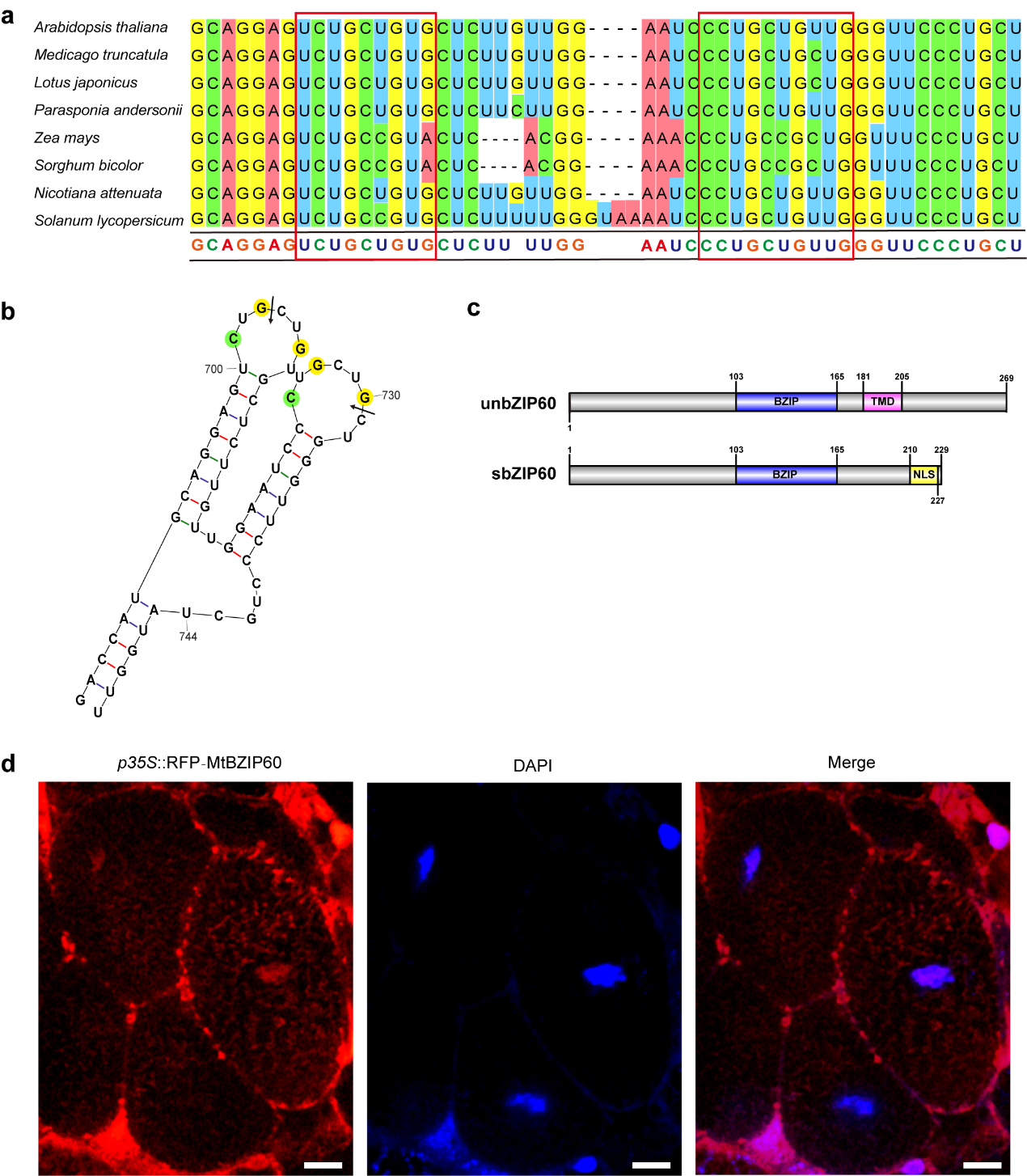


**Figure S4.** Sequence, structure, and subcellular localization features of b*ZIP60* proteins of *M. truncatula*. a) Sequence alignment of *bZIP60* mRNA in *M.truncatula* and other plant species. The alignment was performed using Muscle method. The red frames were used to label the conserve sequences in the loops of secondary structure. b) The predicted secondary structure of recognition site for IRE1, which is composed of a pair of stem loops with conserved bases at three positions in each loop. *MtbZIP60* mRNA splicing is predicted to remove 26 ribonucleotides from the 3'-end. The conserved bases were labeled by solid coloring circles. c) The corresponding primary protein sequence predicted from the unspliced (*unbZIP60*) and spliced (*sbZIP60*) forms of *bZIP60* mRNA. TMD: transmembrane domain (shown in pink); NLS: nuclear location signal (shown in yellow). d) Representative fluorescence of live-cell images showing that RFP-bZIP60 protein localized both on the ER and nucleus, and the nucleus signal was labeled by DAPI (blue) dye. Hand-section samples of three-week-old nodules expressing *Pro35S::RFP-MtbZIP60* were used to view the above subcellular patterns. The rhizobium strain Sm2011-GFP was used for inoculation. Scale bars = 10 μm. At least 60 rhizobial infection cells in a total of 15 nodules were analyzed in three independent experiments.


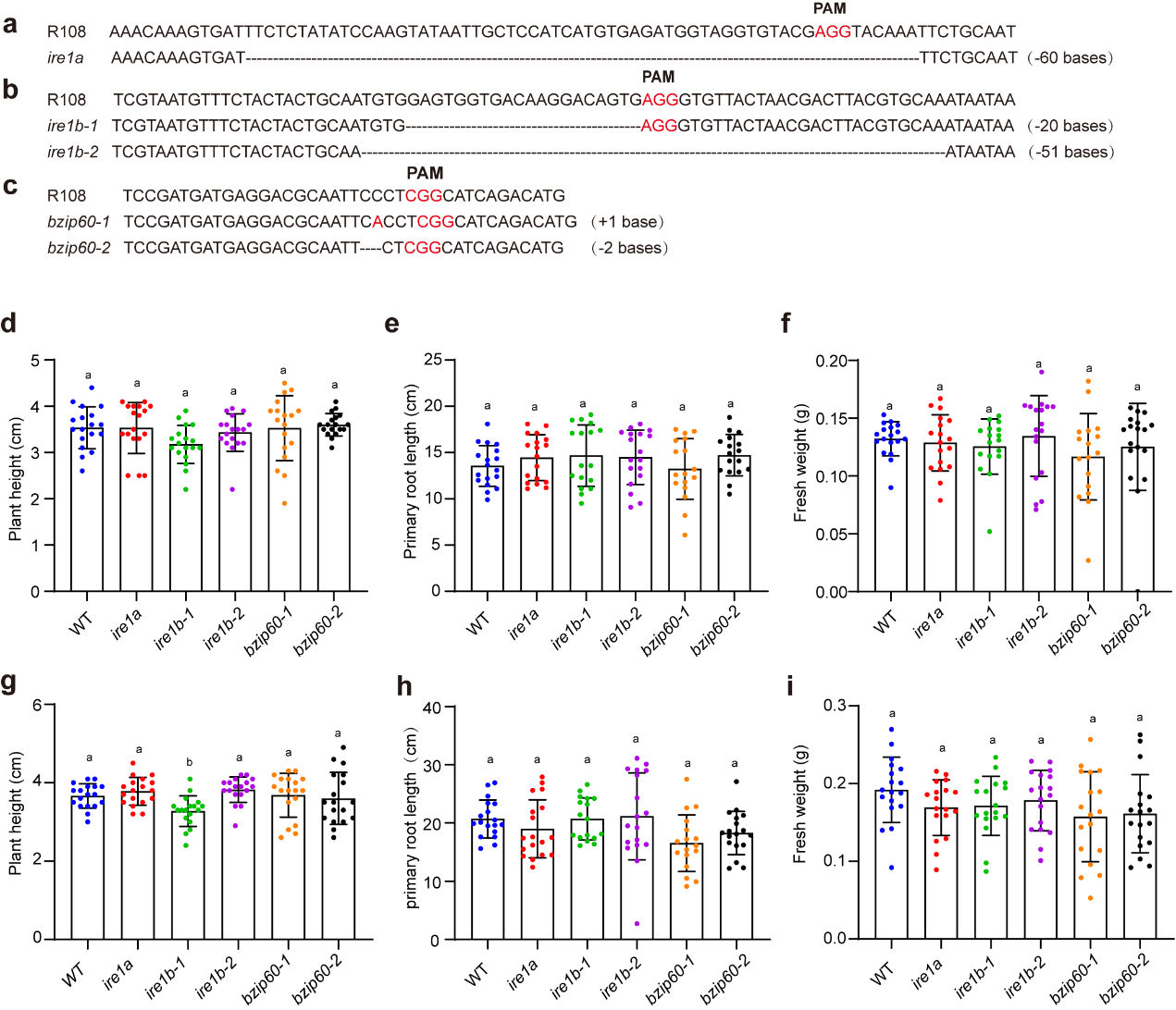


**Figure S5.** *Medicago* UPR-deficient mutant gene-editing information and phenotypic analysis under non-symbiotic conditions. a-c) CRISPR-Cas9-generated deletions in the three UPR genes were characterized in the *ire1a* mutant, a 60-base was deleted (a); in the *ire1b-1* and *ire1b-2* mutants, 20-base and 51-base were deleted, respectively (b); and in the *bzip60-1* mutant, a 1-base insertion was introduced, while in *bzip60-2*, a 2-base was deleted (c). The protospacer adjacent motifs (PAM) highlighted in red. d-i) Quantitative analysis of growth phenotypes included plant height (d, g), primary root length (e, h), and fresh weight of the whole plant (shoots and roots) (f, i) at 12 days (d-f) and 20 days (g-i). Bars and error bars represent the mean and standard deviation, respectively. Statistical analysis was performed using two-way analysis of variance (ANOVA) and post-hoc comparisons (*n* > 16).


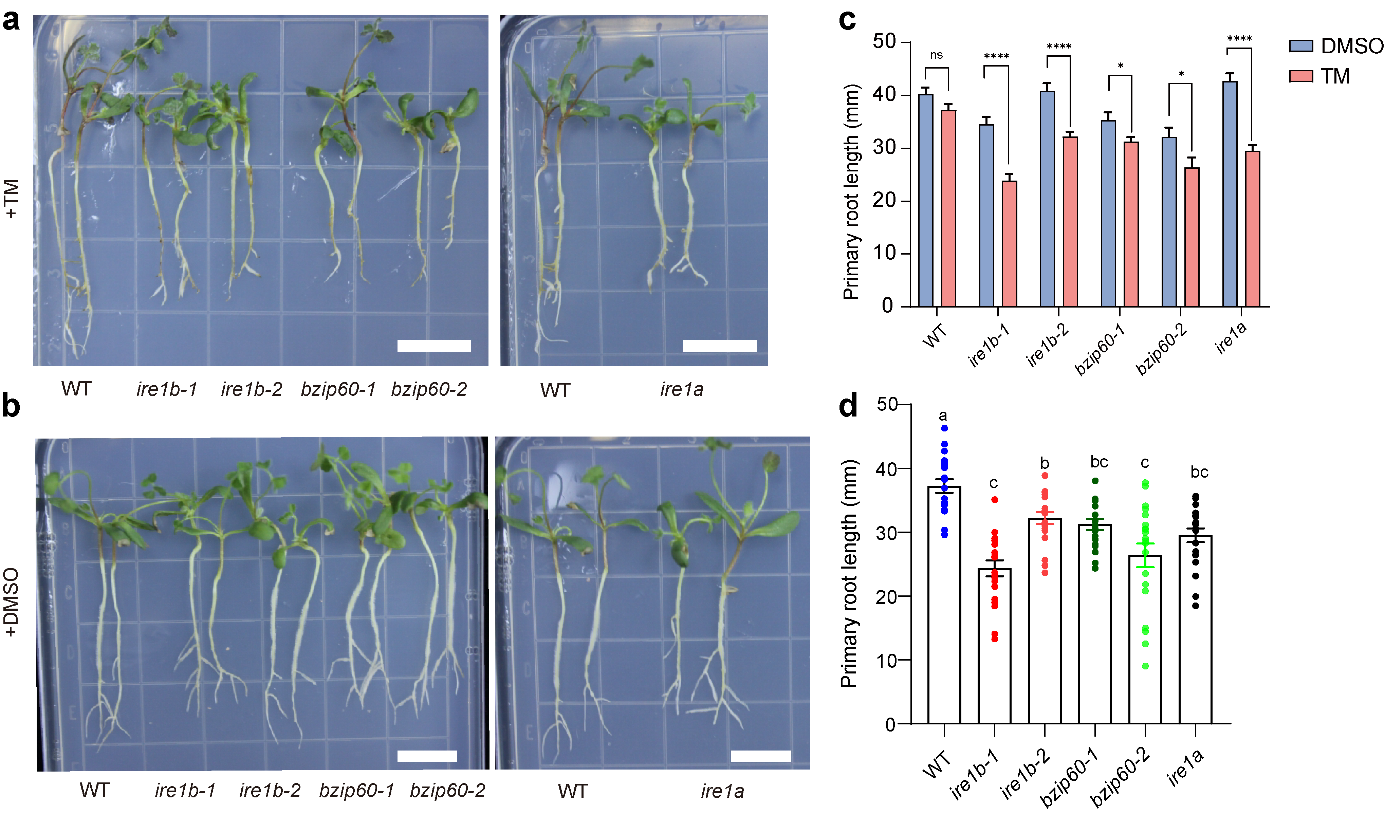


**Figure S6.** Phenotypic analysis of *Medicago* UPR mutants in response to ER stress. a-b) Represent plant growth phenotype under TM treatment (a) and DMSO treatment as control (b), from left to right: WT, *ire1b-1*, *ire1b-2*, *bzip60-1*, *bzip60-2*, *ire1a*. Scale bars = 14 mm. c) Quantitative analysis of primary root length of the above lines under TM treatment or DMSO-treated control. d) Statistic comparison of primary root length in WT and the above mutants after TM treatment. Bars and error bars indicate the mean and the standard deviation, respectively. The statistical analysis was performed using the Student’s t-test in (c) and ANOVA with post-hoc comparisons in (d), respectively. **p* < 0.05, ***p* < 0.01, ****p* < 0.001, *****p* < 0.0001. (*n* >19).


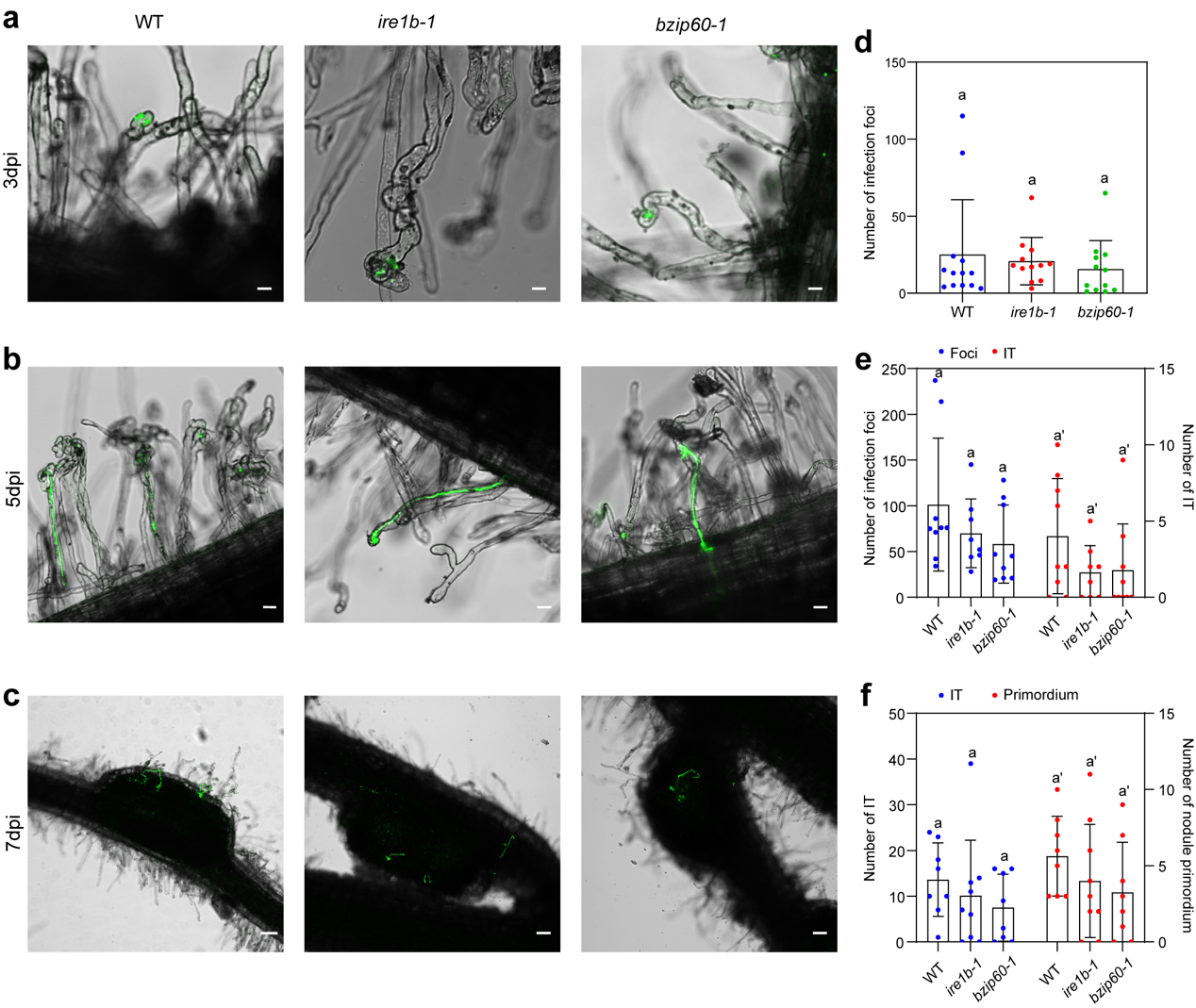


**Figure S7.** Phenotypic analysis of early rhizobial infection events in *Medicago* UPR-deficient mutants. (a-c) Time course analysis of rhizobial infection in wild type (WT), *ire1b-1*, and *bzip60-1*, showing the colonization progression of rhizobia from microcolony formation to ramified cortical infection events at 3 days (a), 5 days (b), and 7 days (c) post-inoculation (dpi) with Sm2011-GFP. Scale bars: 12 μm for (a), 20 μm for (b), and 100 μm for (c). At least 8 plants were analyzed for each time point and genotype. Data are presented as mean ± standard deviation (SD). (d-f) Quantitative analysis of rhizobial infection events in WT and UPR mutants (*ire1b-1* and *bzip60-1*) at 3 (d), 5 (e), and 7 (f) dpi. Statistical analysis was performed using ANOVA with post-hoc comparisons.


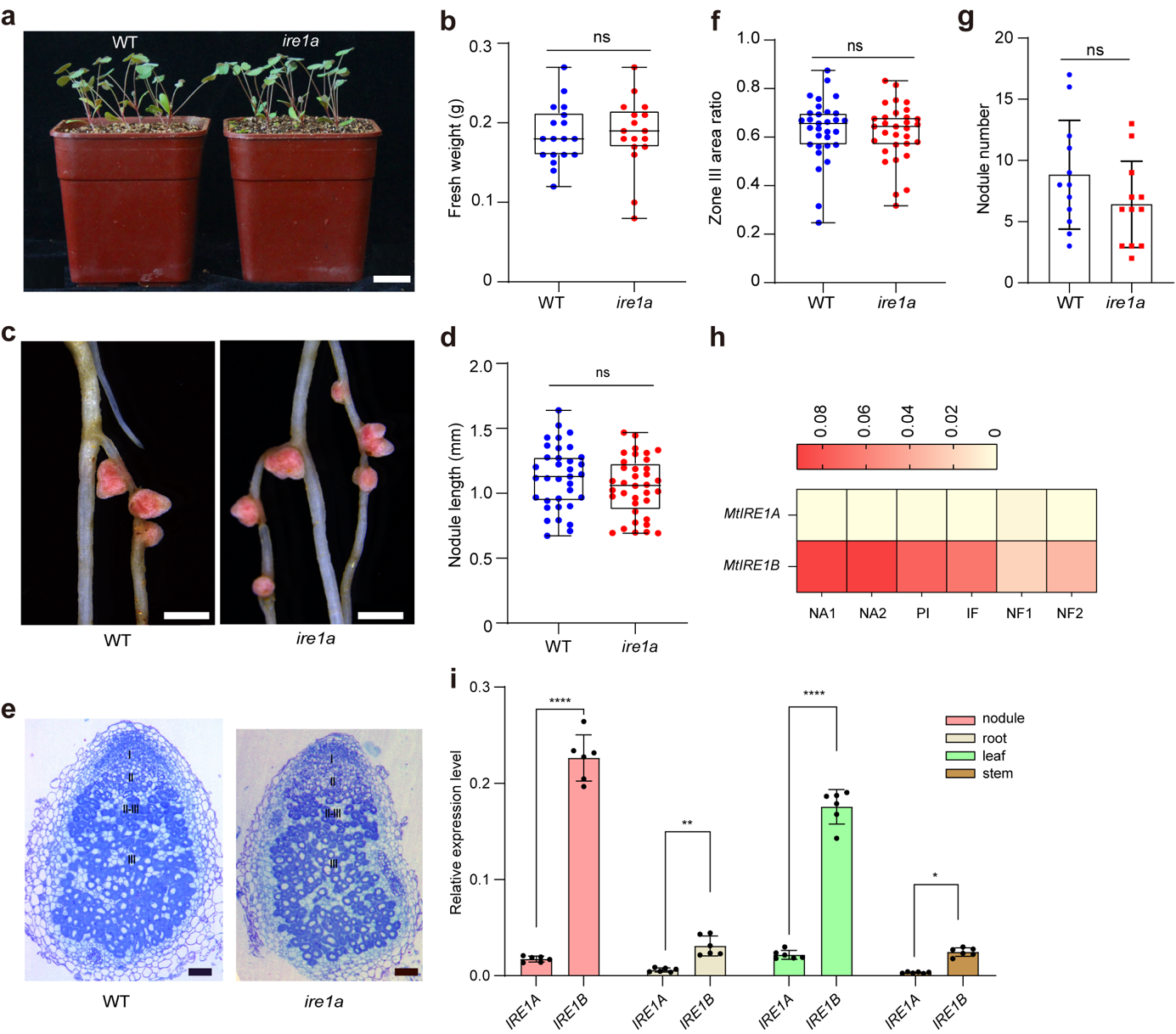


**Figure S8.** Potential functional difference between *IRE1A* and *IRE1B* in the UPR regulation during symbiosis. a) Representative growth phenotypes of WT and *ire1a*; b) Quantitative analysis of the fresh weight in WT and *ire1a* mutant (WT, *n* = 20; *ire1a,* *n* = 19). c) Representative images show the nodule development phenotype in WT and *ire1a* mutant. Scale bars = 2 mm. d) Statistic comparison between nodule length of WT and *ire1a* mutant (WT, *n* = 37; *ire1a,* *n* = 38). e) Representative semi-thin sections of nodules at 14 dpi of WT and *ire1a* mutant; Scale bars = 0.1 mm. f) Quantitative analysis of area proportion of zone III in WT and *ire1a* mutant (WT, *n* = 30; *ire1a,* *n* = 30). g) Quantitative analysis of pink nodule number in WT and *ire1a* mutant at 14dpi (WT, *n* = 12; *ire1a,* *n* = 12). h) The heatmap shows the expression of *MtIRE1A* and *MtIRE1B* in different zones of *Medicago* nodules. The color key indicates the expression level of *MtIRE1A* and *MtIRE1B.* NA1, Nodule apex 1; NA2, Nodule apex; PI, Pre-infection; IF, Infection; NF1, Nitrogen fixation 1; NF2, Nitrogen fixation 2. i) Quantitative analysis of mRNA accumulation of *Medicago* UPR gene *IRE1A* and *IRE1B* in different tissue, (*n* = 6). Bars and error bars in (b, d, f, g, i) indicate the mean and the standard deviation, respectively. Statistical analysis was performed using the unpaired Student’s t-test. ns, not significant; *p* > 0.05.


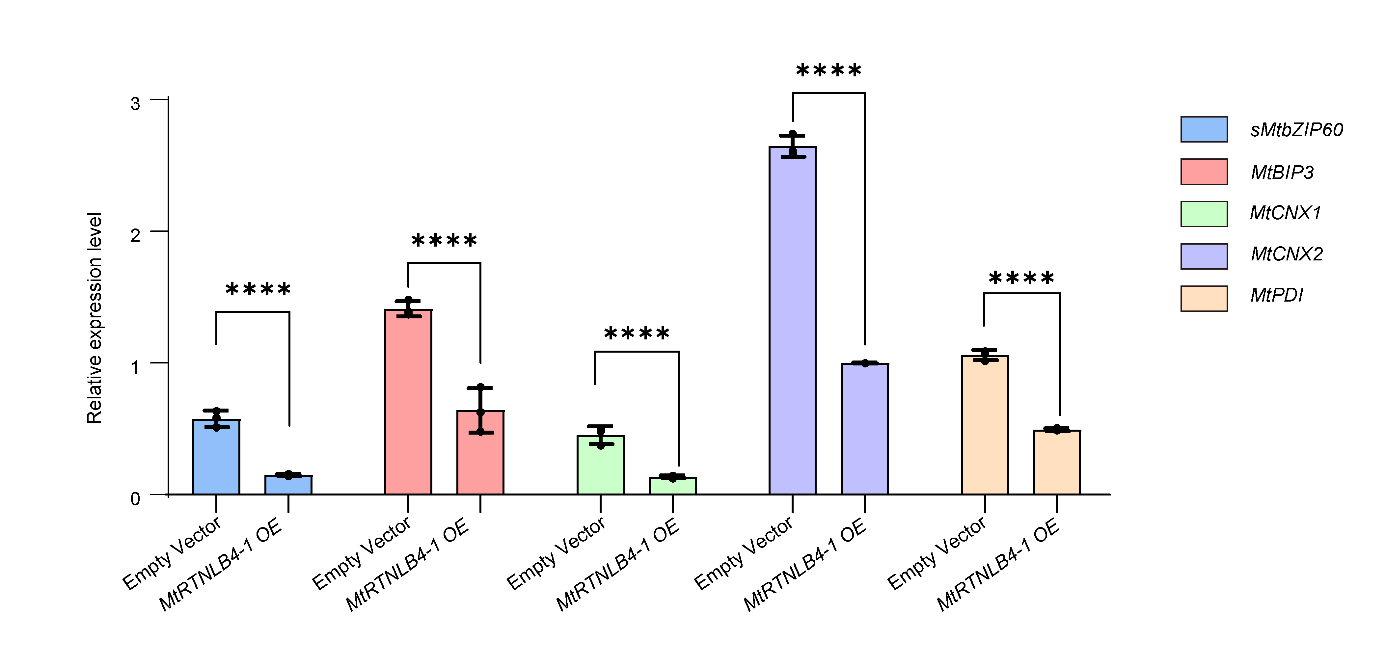


**Figure S9.** Reciprocal feedback regulations exist between ER membrane expansion and UPR signaling. Transcript level of UPR related genes in *MtRTNLB4-1*-overexpressing line was determined by qRT-PCR and the housekeeping gene *MtACTIN11* was used for data normalization. Bars and error bars indicate the mean and the standard deviation, respectively. Statistical analysis was performed using the Student’s t-test, (*n* = 3), *****p* < 0.0001.

**Supplementary movies and table legends**

**Supplementary Movie 1**

SEM micrograph of the serial ultrathin slices for the 3-D reconstruction of infection zone (Zone II) cells, related to Figure S2a;

**Supplementary Movie 2**

3D reconstruction shows that ER in zone II is transforming from sheet lamellar to the tubule ER to enclose symbiosome, related to Figure S2a.

**Supplementary Movie 3**

SEM micrograph of the serial ultrathin slices for the 3-D reconstruction of local symbiosomes distribution in infection zone (Zone II) cells, related to Figure1a;

**Supplementary Movie 4**

SEM micrograph of the serial ultrathin slices for the 3-D reconstruction of local symbiosomes distribution in transition zone (Zone II-III) cells, related to Figure1b;

**Supplementary Movie 5**

SEM micrograph of the serial ultrathin slices for the 3-D reconstruction of local symbiosomes distribution in nitrogen-fixation zone (Zone III) cells, related to Figure1c;

**Supplementary Movie 6**

3D reconstruction shows that symbiosomes in zone II are wrapped by tubular ER, related to Figure1e.

**Supplementary Movie 7**

3D reconstruction shows that symbiosomes in zone II-III are wrapped by expanding tubular ER, related to Figure1f.

**Supplementary Movie 8**

3D reconstruction shows that symbiosomes in zone III are enclosed with connected lamellar ER structures, related to Figure1e.

**Supplementary Movie 9**

SEM micrograph of the serial ultrathin slices for the 3-D reconstruction of infected cells of *MtRTNLB4-1* overexpression group, related to Figure2h.

**Supplementary Movie 10**

3D reconstruction shows the space interaction between symbiosomes and gathered tubular ER structure in infected cells of *MtRTNLB4-1* overexpression group, related to Figure2j.

**Supplementary table1**

List of primer sequences used in this study.
